# Comparative analysis of dose-dependent neurotoxic response to 1-methyl-4-phenyl-1,2,3,6-tetrahydropyridine in C57BL/6 N mice derived from three different sources

**DOI:** 10.1186/s42826-019-0012-2

**Published:** 2019-07-25

**Authors:** Dong-Joo Hwang, Ki-Chun Kwon, Hyun-Keun Song, Kil-Soo Kim, Young-Suk Jung, Dae-Youn Hwang, Joon-Yong Cho

**Affiliations:** 1 Exercise Biochemistry Laboratory, Korea National Sport University, Seoul, 05541 South Korea; 2 Department of Microbiology and Immunology, INJE University College of Medicine, Busan, 47392 South Korea; 3 College of Veterinary Medicine, Kyungpook National University, Daegu, 41566 South Korea; 4 College of Pharmacy, Pusan National University, Busan, 46241 South Korea; 5 Department of Biomaterials Science, College of Natural Resources & Life Science/Life and Industry Convergence Research Institute, Pusan National University, Miryang, 50463 South Korea

**Keywords:** Parkinson’s disease, MPTP, 1-methyl-4-phenyl-1,2,3,6-tetrahydropyridine, C57BL/6Nkorl, Mouse stock

## Abstract

MPTP, 1-methyl-4-phenyl-1,2,3,6-tetrahydropyridine is commonly used to induce nigrostriatal defects to induce parkinsonism and/or parkinsonian syndrome, to replicate the lesions seen in Parkinson’s disease (PD), with use in numerous PD models in mice. It has been suggested that various biological characteristics including strain could result in differing mortality rates, sensitivity to MPTP administration, and reproducibility of lesions in mice, but there is no evidence on the sensitivity of C57BL/6 mice from different origins to MPTP and its associated pathological lesions. In this study, we investigated the magnitude of the dose-dependent response to acute MPTP administration in C57BL/6NKorl mice and two commercialized C57BL/6 stocks derived from the United States and Japan. We measured biological features (body weight, temperature, and composition), nigrostriatal neurotoxic responses (dopamine levels, tyrosine hydroxylase enzymes, and protein carbonylation) and motor function. In results, the three different C57BL/6 stocks exhibited similar overall neurotoxic response and locomotor impairment which increased in a dose-dependent manner with acute MPTP administration (10 mg/kg, 20 mg/kg, and 30 mg/kg, all with external heat support), although some of these differences were not significant. In conclusion, this study provides scientific evidence that C57BL/6NKorl mice can be used as an alternative animal model for practical and targeted PD research.

## Introduction

Parkinson’s disease (PD) is a progressive neurodegenerative disorder. Clinically, its primary symptoms are motor symptoms such as resting tremor, bradykinesia, rigidity, and postural instability. As the disease worsens, non-motor symptoms such as emotional problems, depression, and dementia increase slowly over time [1]. The cause of PD and effective treatments remain unknown, but the aforementioned motor deficits are attributed primarily to the loss of dopaminergic neurons (DA) in the nigrostriatal pathway, a region of the midbrain, caused by genetic or environmental factors [1, 2]. Thus, PD researchers are investigating the pathogenesis and mechanisms underlying the degeneration of DAs.

To this end, rodent models for PD, particularly C57BL/6 mice, have been widely used to unravel various pathological events and explore therapeutic mechanisms, although humans and primates are the gold standard of PD research [3, 4]. Over the years, with numerous efforts to develop a PD model in mice, the exogenous administration of a variety of neurotoxic materials such as 6-hydroxydopamine, paraquat, rotenone, and 1-methyl-4-phenyl-1,2,3,6-tetrahydropyridine (MPTP) have been used to induce DA loss to replicate PD symptoms, collectively called parkinsonism and/or parkinsonian syndrome [5–7]. Among the various pharmacological PD models, the MPTP-induced PD model has been most commonly used for several reasons: similar clinical symptoms to those in patients with PD, no requirements for experimental technology, and reliable and reproducible lesions in the nigrostriatal dopaminergic pathway [3, 8].

In terms of MPTP-induced mouse PD models, it has been suggested that biological characteristics including strain, gender, age, and body weight could influence mortality rates, sensitivity to MPTP administration, and reproducibility of lesions in C57BL/6 mice, as discussed in detail previously [3, 4, 9]. However, there are no data on MPTP sensitivity and pathological lesion reproducibility between C57BL/6 mice derived from different origins at a primary phenotypic and molecular level, especially for C57BL/6Nkorl, a novel mouse stock recently developed in the C57BL/6 strain background by the National Institute of Food and Drug Safety Evaluation (NIFDS) in Korea [10].

Therefore, the present study was conducted to compare the magnitude of the dose-dependent response to acute MPTP administration between C57BL/6 mice derived from different origins (viz., C57BL/6Nkorl stock from Korea, C57BL/6NA stock from the United States, and C57BL/6NB stock from Japan). Our results provide the first scientific evidence that the neuropathological features of C57BL/6Nkorl mice are similar overall to those of other commercially available C57BL/6 mice under MPTP treatments.

## Materials and methods

### Animals

The procedures used for animal experimentation were approved by the Institutional Animal Care and Use Committee of Korea National Sport University (approval number: KNSU-IACUC-2018-10). Male C57BL/6 N mouse stocks (eight-weeks old) were obtained from three different sources (C57BL/6NKorl mice were provided by the Department of Laboratory Animal Resources at NIFDS [Cheongju, Korea], C57BL/6NA were purchased from Orient Bio Inc. [Gyeonggi-do, Korea] and C57BL/6NB was purchased from Japan SLC Inc. [Shizuoka, Japan]). Upon arrival, all animals were randomly divided into four groups (control [*n* = 15], low dose [10 mg/kg, n = 15], moderate dose [20 mg/kg, n = 15], and high dose [30 mg/kg, n = 15]), and acclimatized to the controlled laboratory environment (12:12 h dark–light cycle, 22 ± 2 °C, and 50% relative humidity) with ad libitum access to standard chow diet.

### MPTP administration

MPTP is a neurotoxic chemical that destroys the DA in the nigrostriatal pathway of the midbrain, generating the phenotypic characteristics of PD. Two weeks after acclimation to the laboratory environment, mice assigned to acute MPTP administration regimens were intraperitoneally injected with three different doses (10 mg/kg, 20 mg/kg, and 30 mg/kg) four times every two hours on one day, whereas mice assigned to the control group were injected with saline, all were supported by an external heat pad.

### Evaluation of motor function

Rota-rod test - motor coordination and balance were measured and quantitatively analyzed using a rota-rod (5-lane accelerating rota-rod; JD-A-07, Jeung do, Korea). The animals were pre-adapted for two days to the experimental equipment and exercise method, and the test was performed on the third day. Motor function was evaluated by setting the animal on the equipment, set at 10 rpm, and measuring the elapsed time before the animal loses balance and falls to the floor, with five replicates, and the maximum execution time was set to 300 s [11].

Pole test - after treatment administration, the animals were placed facing upward on top of a vertical pole at an approximate height of 30 cm, and the time taken for animals to descend to the ground was measured in seconds. Motor function was evaluated by measuring the time required for the body and head of animals to turn completely downward (T-turn), and the total time taken to descend to the ground (T-total) [12].

### Biological characteristics

Body weight (BW) and body temperature (BT) of the experimental animals were measured non-invasively using small animal scales and a thermal imaging camera (FLIR C2, FLIR systems, Wilsonville, OR, USA) over the course of the experiment (8 h) at two hour intervals. The body composition of experimental animals was precisely measured by double-energy X-ray absorptiometry before animal sacrifice. The measurement parameters were total body mass (g), fat mass (g), lean mass (g), and fat ratio (% fat).

### Enzyme-linked immunosorbent assay (ELISA)

The dopamine concentration in tissues was analyzed using commercially available ELISA assay kits. The procedure was as follows: to prepare a standard curve, 50 μL of dopamine standard solution was dispensed into a 96-well microplate. Then, 50 μL of homogenized sample supernatant was dispensed into new wells, and 100 μL of horseradish peroxidase (HRP)-conjugate reagent was added, followed by incubation at 37 °C for one hour. After that, 50 μL of Chromogen solutions A and B were dispensed into the 96-well microplates, and incubated at 37 °C for 15 min. The reaction was stopped by adding 50 μL of stop solution to each well, and the absorbance was measured at 450 nm using a microplate reader (HIDEX, Turku, Finland) to calculate dopamine concentration from the standard curve.

### Western blotting

After MPTP administration to experimental animals, 20 mg of brain tissue (substantia nigra and striatum) extracted by autopsy was homogenized by mixing with lysis buffer (ELPIS BIOTECH, EBA-1149), and the supernatant of the obtained sample was used. First, the same amount of protein (20 μg) is electrophoresed by 12% SDS-PAGE gel and transferred to a polyvinylidene fluoride (PVDF membrane soaked in methanol. The membrane was blocked for 60 min at room temperature. And then, the membrane was then incubated overnight at 4 °C with the primary antibodies: Tyrosine hydroxylase (TH, Millipore, 1:1000). After overnight incubation, the membranes were incubated with secondary antibody (1:5000) diluted in 3% BSA solution for 2 h at room temperature. Luminate Forte Western HRP substrate (Millipore, USA) (Molecula Imager ChemiDoc XRS system, Bio-Rad, USA). The protein of interest were identified using Luminate Forte Western HRP substrate (Millipore, USA) for 1 min and the density of each bands was analyzed using an image analysis system (Molecular Imager ChemiDoc XRS system, Bio-Rad, USA).

### Protein carbonyl contents

The immunoblot detection of oxidized protein containing a carbonyl group was conducted using commercially available Oxyblot detection kit (Millipore, Germany). Briefly, 5 μL of purified tissue lysate denatured by adding 12% SDS (A final concentration of 6% SDS) was incubated with 2,4-dinitrophenylhydrazine (DNPH) for 15 min at room temperature, and then 7.5 μL of neutralization solution was added to mixture. The protein samples obtained by the preprocessing process were loaded onto 12% polyacrylamide gels and transferred to the PVDF membrane following routine immunoblot procedures. The membranes were continuously incubated in 1st DNP antibody (Diluted in 1:150 with blocking/dilution buffer) and 2nd antibody (Diluted in 1:300 with blocking/dilution buffer) for 1 h at room temperature with the intermediate wash process. Finally, the oxidized proteins containing carbonyl contents was identified by exposing Luminate Forte Western HRP substrate (Millipore, USA), and the density of the band was analyzed an image analysis system (Molecular Imager ChemiDoc XRS system, Bio-Rad, USA).

### Immunofluorescence staining

After the perfusion of 4% PFA through the heart was performed, the fixed brain tissue was serially sectioned in 40-μm units and stained with the free-floating method. The procedure was as follows. Antigen retrieval of brain tissues was carried out by immersing the sample in a bial containing 0.01 M sodium citrate for 40 min in a water bath at 80 °C, and the blocking was performed with 1% normal serum. After being washed with PBS, primary TH antibody (Diluted in 1:200) was incubated for 48 h at 4 °C and the secondary Alexa 594-conjugated donkey anti-rabbit antibody (Diluted in 1:500) was reacted for 2 h. Finally, the sections of tissue sample were attached to the glass slide and mounted using a mounting medium (H-1000, Vector laboratories, USA). Images of stained brain tissue were obtained using a confocal microscope (TCS SP8, Leica microsystem, Germany). Quantitative analysis of images was performed using LAS AF Lite analysis software.

### Statistical analysis

All data were analyzed using one-way and two-way analysis of variance (ANOVA, followed by Bonferroni post-hoc test to investigate the differences among multiple treatment groups with SPSS version 18.0 (SPSS, Chicago, IL, USA). Data were represented as mean ± standard error of the mean (SEM), and *p* values < 0.05 were considered statistically significant.

## Results

### Dose-dependent changes in biological characteristics

**Fig. 1:**
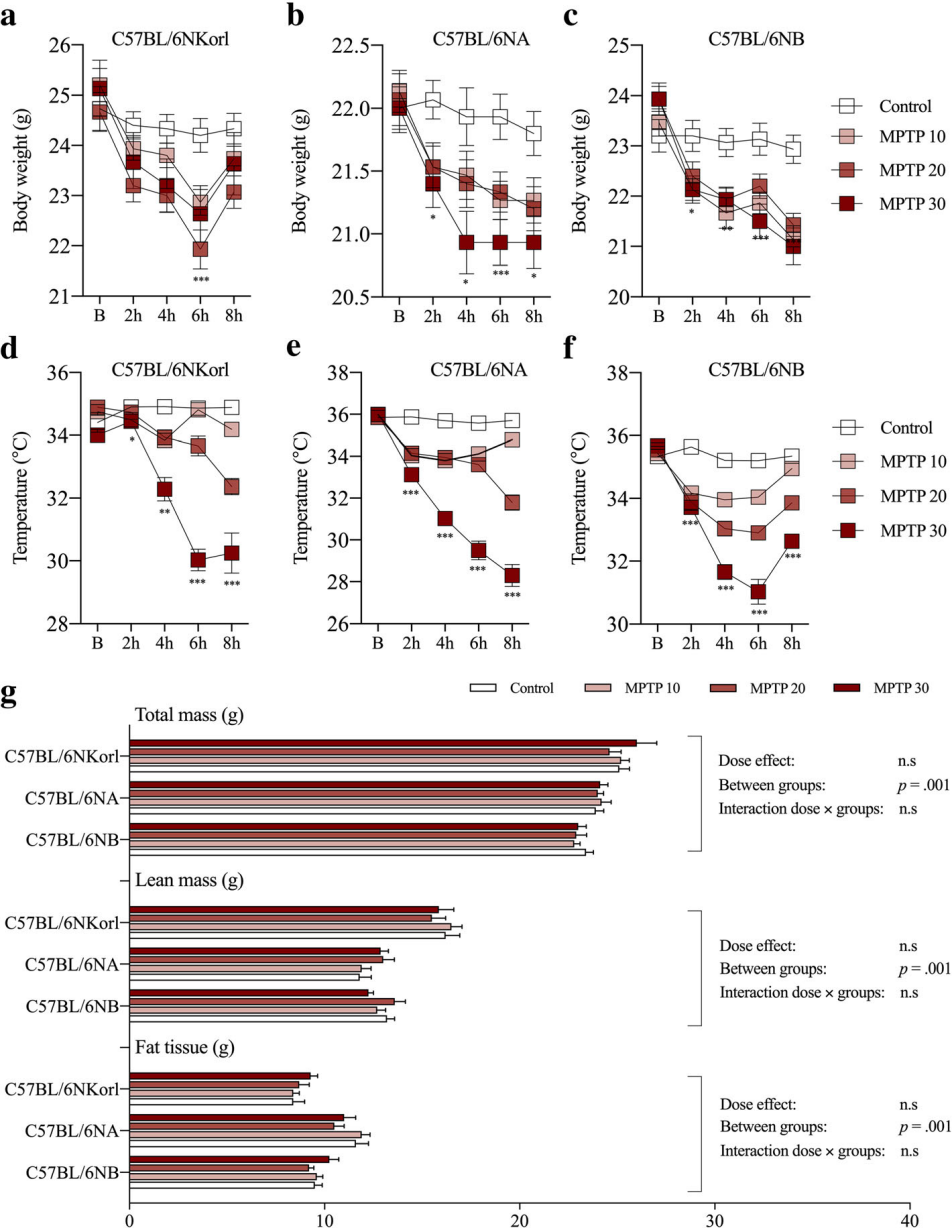
Comparative analysis of physiological responses to acute MPTP administration (four injections at two-hour intervals) among different stocks of C57BL/6 mice. **a**-**c** Dose-dependent changes in body weight and (**d**-**f**) temperature (hypothermic response) over an eight hour period. **g** Distribution and dose-dependent changes in mouse body composition (total mass, lean mass, and fat tissue). Values are presented as mean ± SD; each group consisted of 6–15 mice. *p < 0.05, **p < 0.01, and ***p < 0.001 compared to control group

The dose-dependent changes in BW and BT between the experimental groups are shown in Fig. 1. C57BL/6NKorl, C57BL/6NA, and C57BL/6NB mice showed a dose-dependent rapid decrease in BW and a hypothermic effect of MPTP administration except for unexpected physiological response and a temporary recovery of BW of C57BL/6NKorl. And these phenotypic changes were recovered to normal levels just before sacrifice (seven days following MPTP administration), although the average BW of C57BL/6NKorl mice was slightly higher than the other mouse stocks at the resting state before MPTP administration (Fig. 1g, 24.93 ± 1.6 g in C57BL/6NKorl, 22.05 ± 0.6 g in C57BL/6NA, and 23.63 ± 1.1 g in C57BL/6NB).

We assessed the dose-dependent changes in body compositions of experimental animals seven days after the final MPTP administration compared to the control group (Fig. 1g). Body composition was not significantly different between groups. The slightly higher BW of C57BL/6NKorl mice appears to be due to an altered distribution ratio between lean mass and fat mass (Fig. 1g).

### Nigrostriatal dopaminergic depletion by acute MPTP administration

**Fig. 2:**
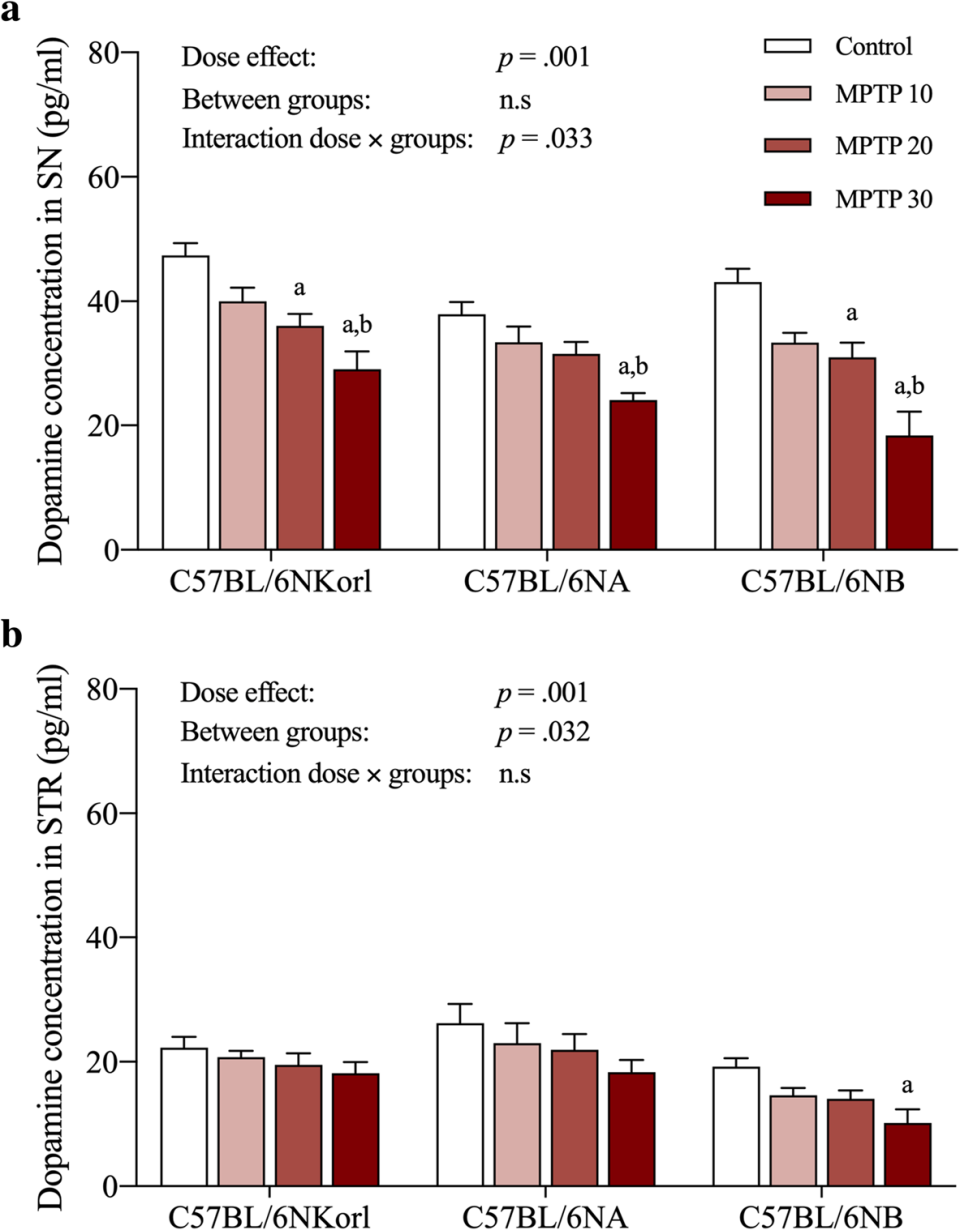
Comparative analysis of nigrostriatal dopamine susceptibility to acute MPTP administration among different stocks of C57BL/6 mice. **a** Dose-dependent changes in dopamine concentration in the substantia nigra (SN) and (**b**) striatum (STR) regions. Values presented are mean ± SD; each group consisted of 5–6 mice. Different letters indicate a significant difference (*p* < 0.05) among treatments within mouse stocks (a denotes statistical significance compared to control group, b denotes statistical significance compared to MPTP 10 group, and c denotes statistical significance compared to MPTP 20 group)

As the dopamine level is a key indicator of neuropathological features in PD models, the level of dopamine in the brain after acute MPTP administration was determined by ELISA (Fig. 2). Dopamine levels in the substantia nigra (SN) and striatum (STR) regions of C57BL/6NKorl, C57BL/6NA, and C57BL/6NB mice showed a tendency to linearly decrease in a dose-dependent manner with MPTP administration compared to the control group, although most of these differences were not significant (Fig. 2).

### Nigrostriatal neurotoxicity after acute MPTP administration

**Fig. 3:**
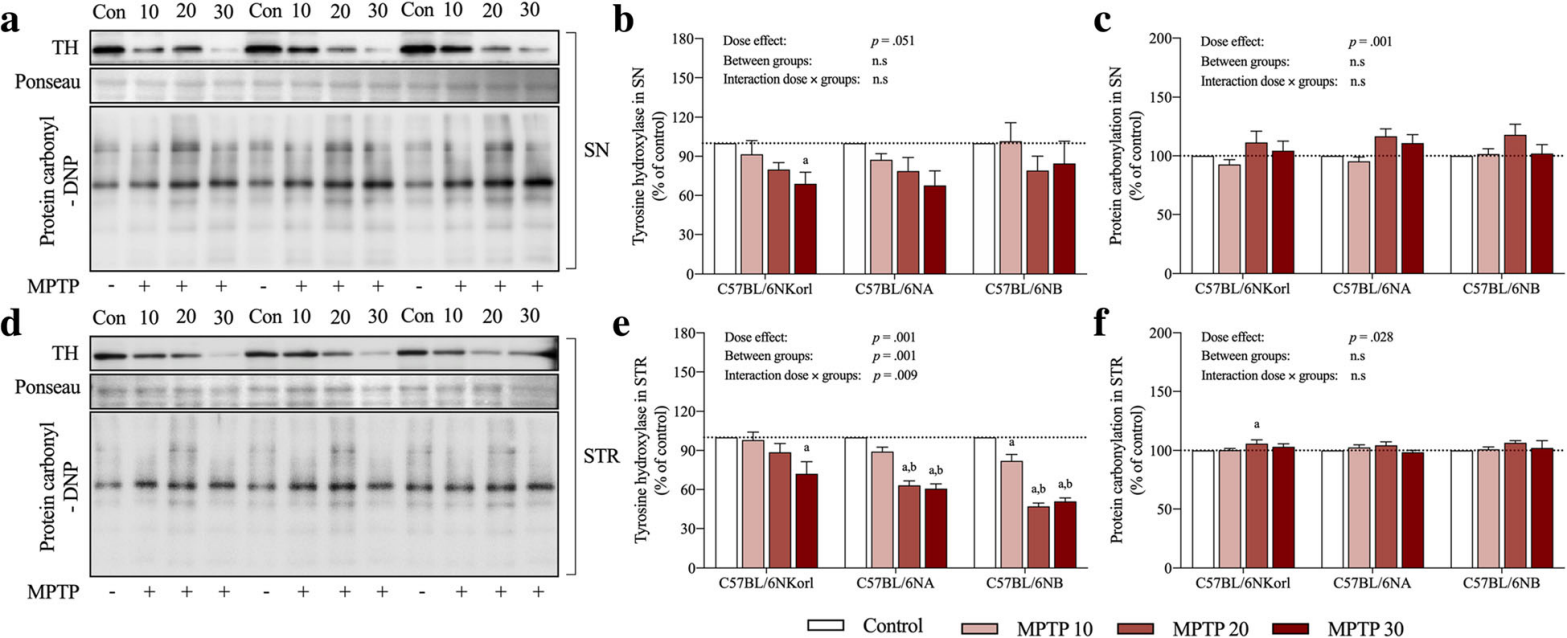
Comparative analysis of the nigrostriatal neurotoxicity and protein oxidation from acute MPTP administration among C57BL/6 mice stocks. **a** and **d** Representative western blots showing levels of tyrosine hydroxylase (TH) expression and protein carbonylation, respectively. **b**, **c**, **e**, and **f** Densitometric analyses of blots normalized for density of Ponseau staining in the SN and STR tissues of different C57BL/6 stocks. The dashed line indicates the level of the control group. Values presented are mean ± SD; each group consisted of six mice. Different letters indicate a significant difference (*p* < 0.05) among treatment groups within mouse stocks (a denotes statistical significance compared to control group, b denotes statistical significance compared to MPTP 10 group, and c denotes statistical significance compared to MPTP 20 group)

**Fig. 4:**
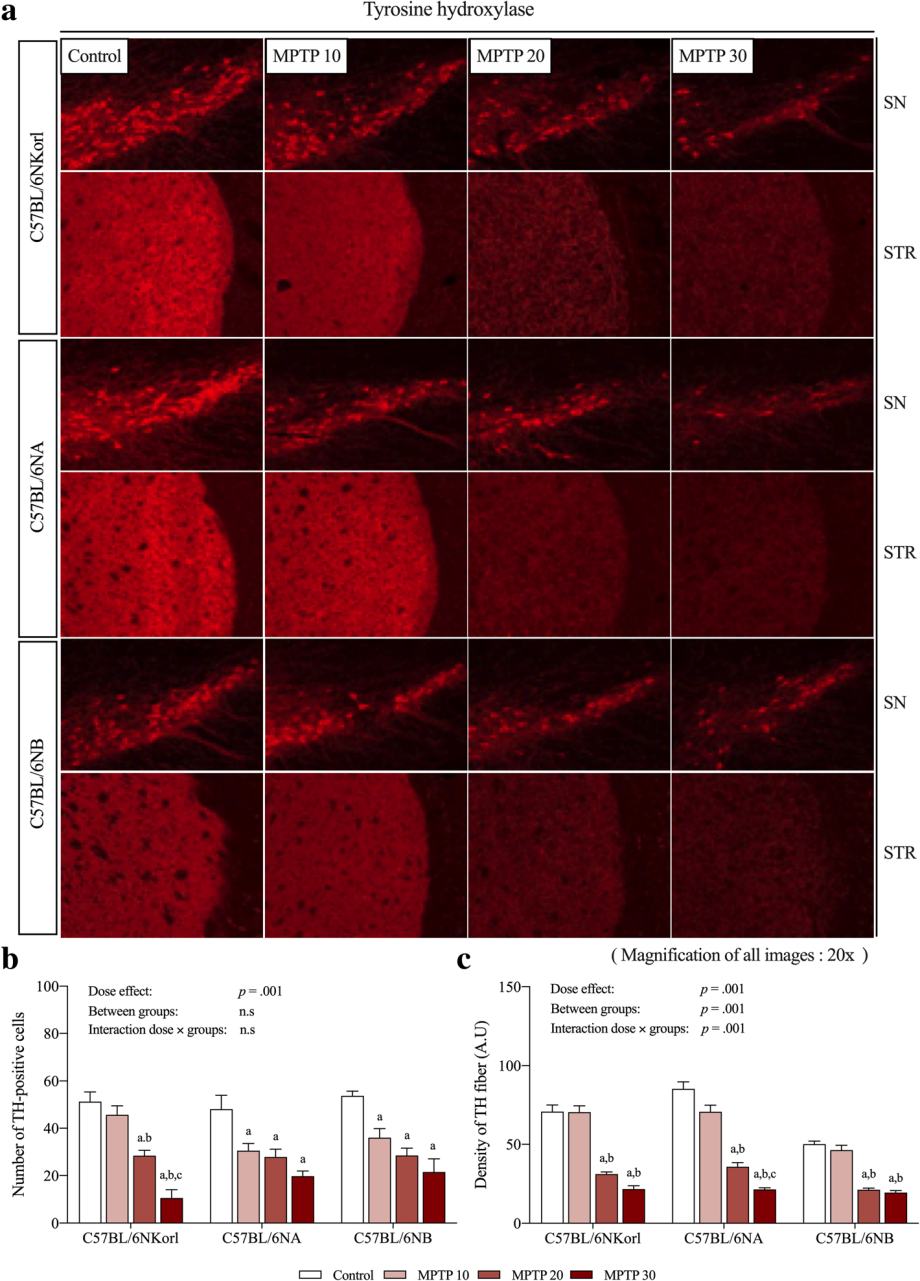
Histopathological and quantitative analysis of tyrosine hydroxylase (TH) expression under acute MPTP administration among different C57BL/6 stocks. **a** Representative immunofluorescence images showing TH in SN and STR regions. Magnification of all images is 20×. **b** and **c** Quantification of TH positive cells and the density of TH positive fibers (arbitrary unit of intensity), respectively, in SN and STR regions. Values presented are mean ± SD; each group consisted of four mice. Different letters indicate significant differences (*p* < 0.05) among treatments within mouse stocks (a denotes statistical significance compared to control group, b denotes statistical significance compared to MPTP 10 group, and c denotes statistical significance compared to MPTP 20 group)

TH is an enzyme involved in dopamine synthesis and is used as a major pathological marker for neurodegenerative changes in PD. In the present study, TH expression in MPTP-treated mice was examined at the protein level (Fig. 3). TH expression of C57BL/6NKorl, C57BL/6NA, and C57BL/6NB mice showed a tendency to decrease in a dose-dependent manner in the SN and STR regions compared to the control group, although there were no significant differences due to high variation except for in SN at 30 mg/kg MPTP of C57BL/6NKorl. We next examined the density and morphological features of TH+ cell bodies and TH+ fibers using immunofluorescence staining (Fig. 4). The dopaminergic cell number of C57BL/6NKorl, C57BL/6NA, and C57BL/6NB mice had a significant linear reduction in a dose-dependent manner at four days after acute MPTP administration compared to the control group, as revealed by stereological counts of TH+ cells and density of TH+ fibers in SN and STR regions (Fig. 4).

Additionally, as an index to evaluate the degree of oxidative stress in the disease state, we examined the level of protein carbonylation following acute MPTP administration (Fig. 3). Protein carbonylation of C57BL/6NKorl, C57BL/6NA, and C57BL/6NB mice showed no tendency to significantly increase in SN and STR regions compared to the control group except in the moderate dose group (20 mg/kg) of C57BL/6NKorl mice.

### Locomotor impairment by acute MPTP administration

**Fig. 5:**
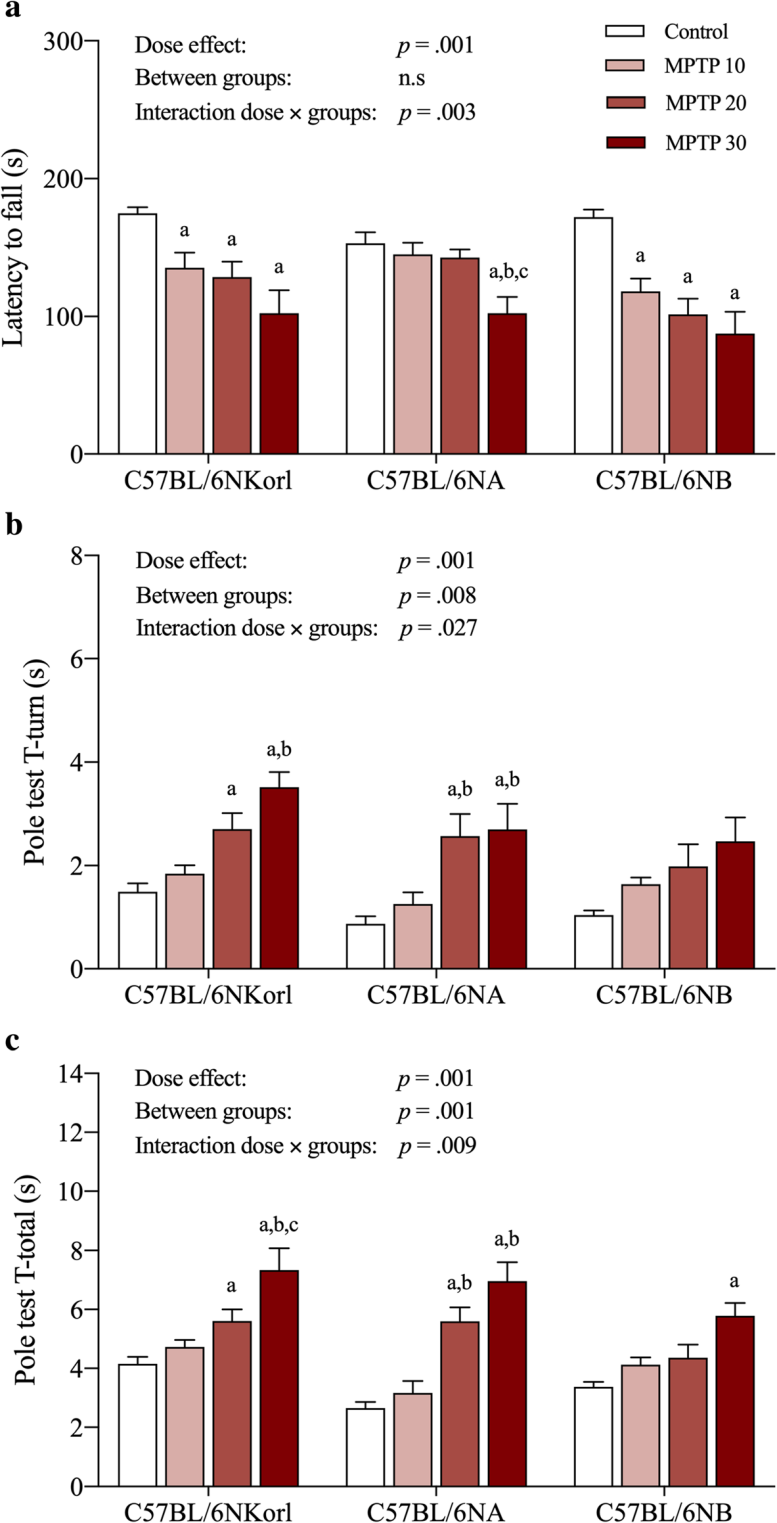
Identification of motor function deficits in response to acute MPTP administration in different C57BL/6 mice stocks. **a** Retention time on rota-rod test and (**b** and **c**) the completion times for T-turn (time for mouse to turn) and T-total (total time taken to descend to the ground), respectively, seven days following the last MPTP injection. Different letters indicate a significant difference (*p* < 0.05) among treatments within mouse stocks (a denotes statistical significance compared to control group, b denotes statistical significance compared to MPTP 10 group, and c denotes statistical significance compared to MPTP 20 group)

Given that PD patients exhibit clinical symptoms such as resting tremor, bradykinesia, rigidity, and postural instability resulting from dopaminergic dysregulation in the brain, we quantitatively examined the level of locomotor impairment in mice by monitoring their performance in a rota-rod test (latency to fall) and pole test (T-turn and T-total) (Fig. 5). Following MPTP administration, C57BL/6NKorl, C57BL/6NA, and C57BL/6NB mice showed a significant decrease in all measures of motor function in a dose-dependent manner with tremor symptoms compared to the control group (Fig. 5). These behavioral dysfunctions are connected to the results of the various molecular biology tests presented above, suggesting that degenerative changes at the cellular level following MPTP administration are linked to the major locomotor impairments generated.

## Discussion

In the current study, we examined the dose-dependent effect of an exogenous neurotoxic substance, MPTP, that has been used to develop an experimental model of PD and to explore its neuropathological mechanisms, in C57BL/6 mice derived from three different origins.

The protoxin MPTP is highly permeable across the blood-brain barrier, penetrates into the brain, and is metabolized to the toxic compound 1-methyl-4-phenylpyridinium through a series of processes that leads to selective degenerative defects in nigrostriatal DAs [5, 8]. Although these pathophysiological mechanisms do not replicate all the features of PD, previous studies have investigated behavior phenotypes and various pathogenic mechanisms using this pharmacologically-induced PD animal model [13, 14], expected to be suitable for PD researches, and detailed discussions are still in progress.

Administering acute MPTP regimens suitable for inducing stable and reproducible lesions of parkinsonism or parkinsonian syndrome showed that MPTP caused a rapid reduction in BW and a hypothermic effect in a dose-dependent manner, and unexpectedly high mortality rates in mice (at MPTP 20 mg/kg or higher) due to profound hypothermia in the absence of external heat support in a preliminary study (data not shown). These results are consistent with previous studies that reported hypothermic responses and sensitivity induced by unknown malfunctional cellular mechanisms, suggesting the need for external heat support in MPTP-induced PD mouse modeling [6].

Body composition was a potential factor contributing to BW and temperature changes, but there were no significant differences in the body composition of mice for a short period after acute MPTP administration. A tendency for higher BW in C57BL/6NKorl mice under MPTP treatment, potentially arising from a different ratio between lean mass and fat mass was identified. Considering previous research [10, 15], this phenotypic difference may be due to the Korl-specific genetic background and further studies are required.

Various pathological mechanisms induced by MPTP administration, such as defects in mitochondrial respiration, oxidative stress, and inflammation cause dopaminergic depletion in the nigrostriatal pathway, which predominantly appears in PD patients [16, 17]. Thus, assessing dopaminergic depletion is an important indicator for establishing a reliable MPTP mouse model of PD. As expected, the expression of TH protein, a rate limiting enzyme involved in dopamine synthesis, and DA concentration in the SN and STR regions showed a tendency to decrease in a dose-dependent manner, and protein oxidation levels appeared to slightly increase to the moderate dose group (20 mg/kg), although most of these differences were not significant. Also, in accordance with the molecular experimental results and previous research [18, 19], we demonstrated that MPTP administration induced significant locomotor impairments in a dose-dependent manner, as validated by the decreased latency to fall and delayed T-turn and T-total values. Taken together, the exceptional case of unexpected toxicity of low dose MPTP suggested in previous study cannot be overlooked [20], these results of acute MPTP administration replicate previous studies reporting MPTP toxicity towards the nigrostriatal pathway and consequent motor dysfunction, and demonstrate the validity of the regimens of MPTP administration and mouse stocks for reliable and reproducible PD models [3, 16, 21].

A limitation of our study was that we could not provide a direct experimental evidences for the effects of metabolic phenotypes of C57BL/6NKorl mice on dose-dependent neurotoxic response, unexpected physiological response and a temporary recovery of BW of C57BL/6NKorl (Fig. 1) shown in this study. Therefore, further studies should carry out comprehensive and logical verification of metabolic response of C57BL/6NKorl mice and its relation to neurotoxicity, which would provide valuable information about the characteristics and commercialization of C57BL/6NKorl mice in the field of laboratory animals.

## Conclusion

In the current In conclusion, the three different C57BL/6 N stocks (viz., C57BL/6NKorl stock from Korea, C57BL/6NA stock from the United states, and C57BL/6NB stock from Japan) exhibited similar pathological lesions towards the nigrostriatal pathway, a region of midbrain and consequent locomotor impairment at multiple doses of acute MPTP administration (all with external heat support), as a preclinical model of PD. Taken together, our results demonstrate the validity of the regimens of MPTP administration and mouse stocks for reliable and reproducible neurotoxic lesions, and provide a scientific evidence that C57BL/6NKorl mice can be used as an alternative animal resource for practical and targeted PD research.

## Data Availability

The authors confirm that the data supporting the findings of this study are available within the article.

## Abbreviations

6-OHDA: 6-hydroxydopamine
BT: Body temperature
BW: Body weight
DA: Dopaminergic neurons
HRP: Horseradish peroxidase
MPTP: 1-methyl-4-phenyl-1,2,3,6-tetrahydropyridine
NIFDS: National Institute of Food and Drug Safety Evaluation
PD: Parkinson’s disease
SN: Substantia nigra
STR: Striatum
TH: Tyrosine hydroxylase
